# Instructions matter: a comparison of baseline conditions for cognitive emotion regulation paradigms

**DOI:** 10.3389/fpsyg.2014.00347

**Published:** 2014-04-28

**Authors:** Kersten Diers, Fanny Weber, Burkhard Brocke, Alexander Strobel, Sabine Schönfeld

**Affiliations:** Department of Psychology, Technische Universität DresdenDresden, Germany

**Keywords:** emotion regulation, baseline condition, instruction, amygdala, fMRI

## Abstract

The choice of a meaningful baseline condition is a crucial issue for each experimental design. In the case of cognitive emotion regulation, it is common to either let participants passively view emotional stimuli without any further specific instructions or to instruct them to actively attend to and permit any arising emotions, and to contrast one of these baseline conditions with a regulation condition. While the “view” strategy can be assumed to allow for a more spontaneous emotional response, the “permit” strategy may result in a more pronounced affective and cognitive response. As these conceptual differences may be associated with differences both in subjective emotional experience and neural activation, we compared these two common control conditions within a single functional magnetic resonance imaging (fMRI) experiment, during which participants were instructed to either passively view a set of unpleasant and neutral pictures or to actively permit any emotions arising in response to the unpleasant pictures. Trial-by-trial ratings confirmed that participants perceived the unpleasant pictures as more arousing than the neutral pictures, but also indicated higher subjective arousal during the “permit negative” as compared to the “view negative” and “view neutral” conditions. While both the “permit negative” and “view negative” conditions led to increased activation of the bilateral amygdala when contrasted with the passive viewing of neutral pictures, activation in the left amygdala was increased in response to the “permit” instruction as compared to the “view” instruction for unpleasant pictures. The increase in amygdala activation in both the “permit” and “view” conditions renders both strategies as suitable baseline conditions for studies of cognitive emotion regulation. Conceptual and activation differences, however, indicate that these two variants are not exchangeable and should be chosen depending on the experimental context.

## INTRODUCTION

In a typical fMRI experiment in the field of cognitive emotion regulation, participants are confronted with an emotional stimulus and are asked to regulate their affective responses using a particular strategy. This may involve changing one’s attentional focus, the way to interpret the emotional stimulus, or one’s visible response toward it (Gross, 1998). Across a growing number of studies, a general pattern of results has emerged, which usually involves increased activation in a cognitive control network and decreased activation in one or more affective target regions, most notably, although not exclusively, the amygdala (Kalisch, 2009; Ochsner et al., 2012). Still, heterogeneity of samples, stimuli, and experimental designs limits the comparability across experiments, meta-analytic integration, and, ultimately, translation into applied domains. To address this issue, one strategy is to decompose the complex construct of emotion regulation into an ensemble of sequential elementary processes, which may be better suited for rigorous experimental investigation (Ochsner and Gross, 2005). A complementary approach is to explore and systematically vary the defining features of cognitive emotion regulation paradigms. This is an aim of the present study.

Among the many variables relevant for the design of emotion regulation paradigms, the instructions on how to regulate emotions are of critical importance. They are the main means of experimental manipulation for emotion regulation strategies such as reappraisal, detachment, or intensification. They all have in common that they pose high cognitive demand in terms of, e.g., verbal abilities and self-regulatory capacities. Consequently, the success of the experimental manipulation will highly depend on the degree of participants’ understanding of and adherence to the instructions. While several reports already have investigated the efficacy of different regulatory strategies (McRae et al., 2010; Kanske et al., 2011), the issue of selecting a valid control or baseline condition has received less attention so far. One study by Schaefer et al. (2002) demonstrated greater amygdala activation for an instruction to actively maintain the emotional response than for an instruction to passively view an emotional stimulus; this effect, however, was most pronounced not during, but after the presentation of the emotional stimulus. The selection of an appropriate baseline condition, however, is as important as the choice of an effective treatment condition, since fMRI usually involves contrasting two or more experimental conditions. Due to this relative nature of fMRI effects, the conclusions that can be drawn from a given study will depend not only on the treatment condition, but also on its counterpart, the control or baseline condition, against which the effects of interest are compared.

Several variants of control or baseline conditions have been employed in recent studies on cognitive emotion regulation: frequently, participants are instructed to simply view emotional stimuli and respond naturally without regulation, which is commonly referred to as “passive viewing” (Harenski and Hamann, 2006; Mak et al., 2009). Alternatively, participants may be asked to specifically attend to the emotional stimulus without referring to the emotional reaction [“attentive viewing”; e.g., Urry et al. (2006) and Taylor et al. (2003)]. Finally, another common choice is to instruct participants to “Look at stimulus directly and permit feeling your emotions,” i.e., to actively permit any emotions arising in response to the emotional stimulus [“emotional allowance”; e.g., Wager et al. (2008) and Walter et al. (2009)]. In being more detailed than the passive viewing instruction, this instruction is similar to the attentive viewing instruction; it puts, however, more emphasis on the experiential aspect of the emotional response than on the emotional stimulus *per se*.

All of the above instructions share the goals of maximizing the effect of the emotion induction and providing an interpretation as unambiguous as possible. However, they are also likely to stimulate different affective and cognitive processes: attentive viewing and emotional allowance, for example, can be assumed to result in a more thorough evaluation of the emotional stimulus and/or response, possibly involving higher cognitive processes such as attention or language; they may, however, differ with regard to an internal (emotional allowance) vs. external (attentive viewing) focus of attention. Passive viewing, due to its unspecific nature and less amount of experimental control, may allow for a more spontaneous emotional response, possibly resulting in a stronger influence of individual differences or situational factors. Altogether, it is likely that the use of different instructions will result in both quantitative and qualitative differences in subjective affective experience, cognitive processes, and physiological activation.

We therefore investigated the behavioral and neural effects of two different baseline conditions by directly comparing them within one single experiment. Specifically, we chose to compare the emotional allowance and passive viewing strategies, i.e., the “permit” and “view” instructions, which have frequently been employed as baseline conditions before. We hypothesized that the “permit” instruction would lead to an increase in activation in brain regions subserving the generation of emotional responses, and therefore defined the left and right amygdala as our regions of interest. Apart from that, all other analyses were conducted in an exploratory way, since the contributions of other brain regions to differences between the experimental conditions are not well defined yet.

## MATERIALS AND METHODS

### PARTICIPANTS

Thirty-two volunteers (12 male, age range 18–48 years, age mean ± SD = 24.7 ± 5.2 years) were recruited from the university community and participated in this study. All of the participants were right-handed and did not report any current medical or prior or current neurological or psychiatric illness or treatment. The experimental protocol was approved by the ethics committee of the Technische Universität Dresden. All participants provided written informed consent and received financial compensation for their time and effort.

### EXPERIMENTAL PARADIGM AND PROCEDURE

In our experimental task, participants were asked to either passively view a set of unpleasant and neutral pictures or to actively permit any emotions arising in response to a set of unpleasant pictures. We did not include a “permit neutral” condition in the experiment due to economical reasons and an assumed lack of validity of this condition, since we assumed that there would likely be no initial emotional reaction for neutral stimuli that might be the target of subsequent emotional allowance.

Written instructions gave an explanation and some examples of these modes of processing: for the “view neutral” and “view negative” conditions, participants were asked to simply view the pictures. We intentionally refrained from giving any further specific instructions in order to elicit a maximally spontaneous emotional reaction. For the “permit negative” condition, participants were asked to take a close look at the picture and permit any emotions that might arise as a result. They were encouraged, but not obliged, to imagine immediately witnessing the depicted situation, and asked not to re-interpret the situation, to voluntarily intensify their emotions, or to distract themselves.

To ensure understanding of the instructions and familiarity with the procedure, all participants underwent a training session outside the MR scanner, which took about 10–15 min and consisted of 16 trials. After completion, they were asked to explain what they did; if this report was incompatible with prior instructions or participants reported difficulties with the task, instructions were explained for a second time, and participants were asked to do another training session. All stimuli used in the training session were different from those used in the main experiment.

The subsequent fMRI measurement lasted for approximately 55 min. In the beginning of the session, about 5 min were spent with preparatory technical scans, which allowed the participants to accommodate themselves with the scanning environment. Two experimental runs followed with a duration of 15 min each, and afterwards an anatomical scan was acquired, which lasted approximately 7 min. Finally, an emotional reactivity experiment took place, which is not the focus of the present study. After completing the scanning session, participants were asked to fill in a questionnaire regarding their subjective experience, and were debriefed about the experiment. Several participants also completed a number of additional questionnaires aimed at investigating individual differences, or took part in a second session encompassing another emotion regulation experiment; both will be the subject of a subsequent report.

Similar to previous studies (McRae et al., 2010; Kanske et al., 2011), we used an event-related design (in contrast to a block design) in order to be able to avoid multiple repetitions of stimuli of the same experimental condition and possibly ensuing increased habituation effects. We further used a relatively slow rate of stimulus presentations in order to allow for sufficient time to implement a processing strategy, and in order to prevent the introduction of additional task-switching demands, which are to be expected if changes between experimental conditions occur at a rapid pace.

In particular, each of the two experimental runs consisted of 30 trials: 10 trials each for “permit negative” and “view negative” and another 10 trials for “view neutral.” At the beginning of each trial, a picture was presented for 8000 ms. During the initial 2000 ms of this period, a semi-transparent overlay was presented across the center of the picture, which contained, as a single word, the instruction for either the “view” or the “permit” condition. As detailed above, “permit” was presented only for negative pictures, while “view” was presented for both neutral and negative pictures. Subsequently, participants were asked to give a rating of their momentary subjective arousal, which lasted for 3000 ms. The rating was intended to reflect the critical role of the amygdala in arousal regulation (Hamann, 2012). Therefore, it asked “How aroused do you feel at the very moment?” and participants responded by moving a horizontal slider to a position between the two extremes “very much aroused” and “not at all aroused.” Following the rating, a fixation cross was presented for a period of 12000 ms. This extended fixation period was inserted into the trial to allow the return of the BOLD response to baseline levels. After another arousal rating (3000 ms), which was included to assess subjective arousal levels at the end of the fixation period, and a variable interval with a mean duration of 4000 ms, during which a fixation cross was presented, the next trial began. Altogether, the duration of a single trial was, on average, 30 s.

Stimuli were selected from the International Affective Picture System (Lang et al., 2008). We used four sets of negative pictures and two sets of neutral pictures (20 pictures per set), which were matched for content, arousal, and valence, respectively (mean valence of negative images: *V*_1_ = 2.78, *V*_2_ = 2.81, *V*_3_ = 2.67, *V*_4_ = 2.76; mean arousal of negative images: *A*_1_ = 5.74, *A*_2_ = 5.74, *A*_3_ = 5.54, *A*_4_ = 5.64; mean valence for neutral images: *V*_5_ = 5.16, *V*_6_ = 4.98; mean arousal for neutral images: *A*_5_ = 2.86, *A*_6_ = 3.04). Neither the negative, nor the neutral sets of images differed in their arousal [negative: *F*_(3,76)_ = 0.281, *p* = 0.839; neutral: *F*_(1,38)_ = 0.985, *p* = 0.327] or valence [negative: *F*_(3,76)_ = 0.121, *p* = 0.947; neutral: *F*_(1,38)_ = 0.902, *p* = 0.348] ratings, and thus were collapsed into two sets of negative and neutral images, which, in contrast, were clearly distinct in their valence [*t*_(118)_ = –16.464, *p* < 0.001] and arousal [*t*_(118)_ = 19.240, *p* < 0.001] ratings. The negative pictures consisted primarily of depictions of animals, bodies, disaster, disgust, injuries, suffering, or violence, while the neutral pictures depicted various scenes, objects and people. In order to rule out any stimulus- or content-related confounds, two sets of negative pictures and one set of neutral pictures were used for one half of the participants, and the other two negative and one neutral sets were used for the other half of the participants. The distributions of faces or others parts of the body, depictions of single persons, depictions of multiple persons, any natural stimuli, or inanimate objects or scenes did not differ between the picture sets. The assignment of the negative pictures to either the “view negative” or “permit negative” condition was counterbalanced across participants. All stimuli were presented onto a back-projection screen located at the rear end of the scanner and were viewed through a mirror attached to the head coil.

### DATA ACQUISITION

Magnetic resonance (MR) imaging was done on a 3 Tesla scanner (Siemens Trio; Siemens Erlangen, Germany), using a 12 channel head coil. Functional (T2^*^) MR images were acquired using an EPI sequence with 42 axial slices (slice thickness 2 mm) per volume (TR 2410 ms; TE 25 ms; flip angle 80°; slice gap 1 mm; field of view 192 mm; matrix size 64 × 64). In addition, anatomical (T1) images were acquired using an MPRAGE sequence that consisted of 176 sagittal slices with a thickness of 1 mm (TR 1900 ms; TE 2.26 ms; flip angle 9°; FOV 256 mm; matrix size 256 × 256).

### DATA ANALYSIS

Imaging data analysis was performed using Matlab 7.4 (MathWorks, Natick, MA, USA) and SPM 8^1^. After discarding the first four volumes of each run, preprocessing consisted of slice-timing correction, motion correction, coregistration of individual functional and anatomical data, spatial normalization of the anatomical images to the MNI template, application of the estimated transformation parameters to the coregistered functional images using a resampling resolution of 2 mm × 2mm × 2 mm, and spatial smoothing of the functional images (FWHM 8 mm).

First-level statistical analysis was performed using a general linear model with three regressors for the experimental conditions “view neutral,” “view negative,” “permit negative.” Two additional regressors of no interest were included to account for possible effects of the early and late ratings and to avoid potential misattribution of these effects to one of the regressors modeling the response to the IAPS pictures. All above regressors were convolved with the canonical HRF. Since the effects of head motion on fMRI time-series are likely more complex than simple linear additive effects, we did not include motion parameters as nuisance parameters in the model in favor of keeping the statistical models as parsimonious and intuitive as possible. The two imaging runs were combined within a single, unifying fixed-effects model of the two runs. Resulting parameter estimates for the contrasts of interest during each run were averaged across runs, submitted to a second-level, random-effects analysis and evaluated using a one-sample *t*-test. Based on our *a priori* hypotheses, we employed two regions of interest, the left and right amygdala as defined by the AAL atlas (Tzourio-Mazoyer et al., 2002; Maldjian et al., 2003), and implemented in the WFU PickAtlas toolbox for SPM^2^. For these analyses, we applied a threshold of *p* = 0.05 family-wise error (FWE) after correction for small volume. For all other analyses, a threshold of *p* = 0.05 FWE across the whole brain was applied. Since voxel-wise analyses are necessarily limited in integrating data across a predefined anatomical structure, we additionally obtained summary measures for the left and right amygdala. For this purpose, we extracted parameter estimates for all experimental conditions from the individual first-level analyses using the marsbar toolbox^3^, and further analyzed these data using *t*-tests for dependent samples.

Behavioral data analysis was performed using R 3.0.1^4^, and consisted of a one-way ANOVA with subsequent *post hoc* testing using *t*-tests for dependent samples. We further conducted an exploratory correlation analysis of subjective arousal ratings and changes in amygdala activation using Spearman’s rho. All analyses, except those conducted within SPM8, were conducted as two-tailed tests.

## RESULTS

### BEHAVIORAL DATA ANALYSIS

**FIGURE 1:**
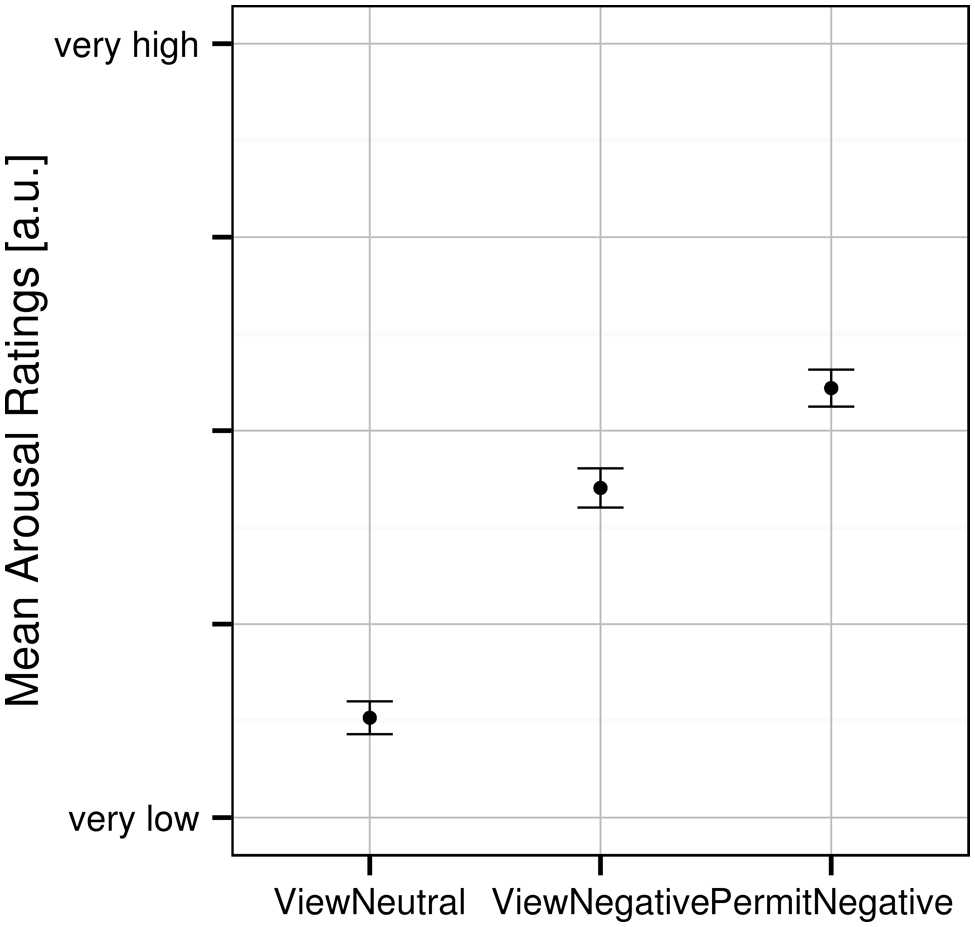
**Mean subjective arousal ratings for the three experimental conditions.** Error bars indicate SEM.

A one-way ANOVA revealed a significant effect of experimental condition [*F*_(2,62)_ = 194.548, *p* < 0.001] on the subjective arousal ratings. Planned *t*-tests revealed significant differences for all pairs of experimental conditions (**Figure 1**): participants rated their arousal highest during the “permit negative” condition as compared with both “view negative” [*t*_(31)_ = 7.031, *p* < 0.001] as well as “view neutral” [*t*_(31)_ = 16.586, *p* < 0.001] conditions. In addition, subjective arousal in the “view negative” condition was greater than in the “view neutral” condition [*t*_(31)_ = 13.621, *p* < 0.001].

### REGIONS OF INTEREST ANALYSIS

**FIGURE 2:**
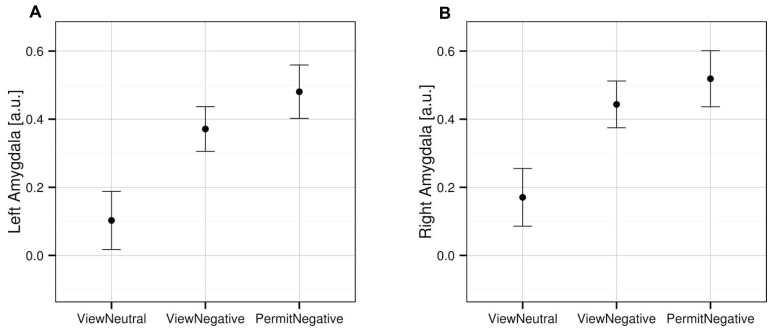
**Mean activation in the left (A) and right (B) amygdala for the three experimental conditions, based on extracted values from the anatomically defined ROI volumes (see Materials and Methods).** Error bars indicate SEM.

**FIGURE 3:**
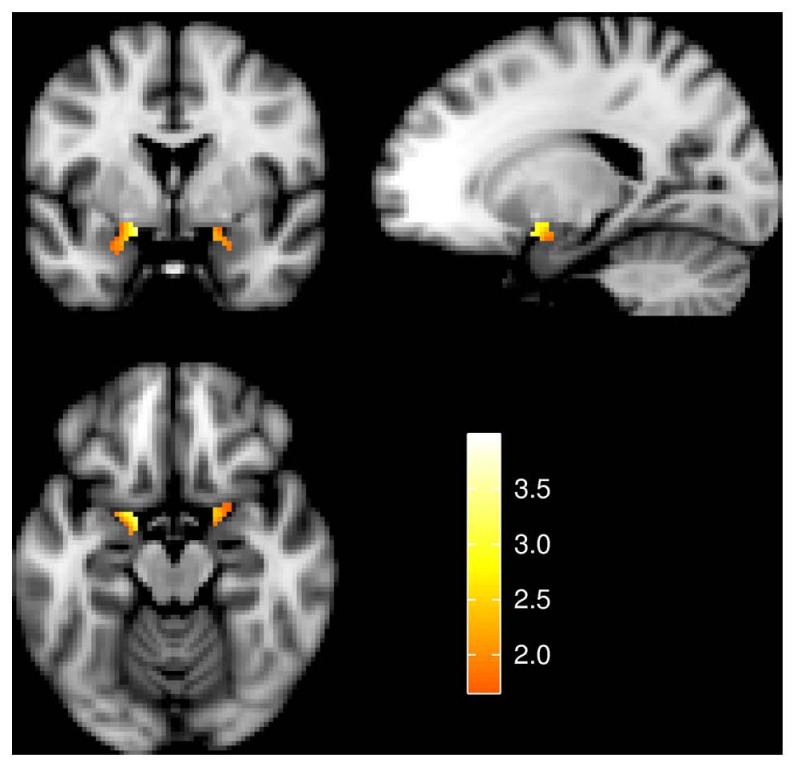
**Amygdala activation for the effect of “permit negative” > “view negative.”** Slices are at *y* = 0 (top left), *x* = -18 (top right), and *z* = -16 (bottom left). Image left is anatomical left. All statistical images represent *t*-values and are thresholded at 1.65 ≤ *t* ≤ 4.00.

An analysis limited to the left and right amygdala yielded significant activation increases in the “view negative” as compared to the “view neutral” condition (**Table 1**), and this effect was also present in an analysis of extracted values [left amygdala: *t*_(31)_ = 3.709, *p* = 0.001; right amygdala: *t*_(31)_ = 4.594, *p* < 0.001; see **Figure 2**]. Similar activation increases were observed for the “permit negative” vs. “view neutral” comparison in the left and right amygdala (**Table 1**). The analysis of extracted values also showed this effect for both the left [*t*_(31)_ = 4.370, *p* < 0.001] and right [*t*_(31)_ = 4.178, *p* < 0.001] amygdala (**Figure 2**). Finally, we found a cluster of greater activation during the “permit negative” condition as compared to the “view negative” condition in the left amygdala (see **Table 1**; **Figure 3**). This was confirmed by an additional analysis of extracted values, which revealed significant effects for the left amygdala [*t*_(31)_ = 2.079, *p =* 0.046], but not right amygdala [*t*_(31)_ = 1.168, *p* = 0.252].

**Table 1:** Results for statistical contrasts between experimental conditions.

Label	H	BA	*t*	*p*	size	*x*	*y*	*z*
**View negative > view neutral**
Middle temporal gyrus	R	19	13.80	<0.001	18612	48	-72	0
Inferior frontal gyrus	R	48	10.04	<0.001	1163	40	14	28
Pallidum	R	-	9.65	<0.001	2035	18	4	8
Supplementary motor area	R	8	7.94	<0.001	292	8	24	48
Thalamus	L	-	7.70	<0.001	60	-22	-30	8
Temporal Pole	L	38	7.67	<0.001	194	-40	20	-16
Inferior frontal gyrus	R	45	7.65	<0.001	137	50	28	2
Inferior frontal gyrus	L	48	7.45	0.001	527	-38	10	22
Superior frontal gyrus	L	6	6.99	0.002	115	-20	8	68
White matter	L	-	6.59	0.005	29	-28	-16	-6
Brain stem	L/R	-	6.56	0.005	49	0	-32	-28
Inferior frontal gyrus	R	45	6.43	0.007	28	54	40	2
Middle frontal gyrus	R	6	6.41	0.008	60	34	2	54
Amygdala*	L	-	4.70	0.001	122	-30	-4	-20
Amygdala*	R	-	4.91	0.001	215	26	-2	-14
**Permit negative > view neutral**
Fusiform gyrus	L	37	12.86	<0.001	19312	-40	-52	-12
Inferior frontal gyrus	R	38	9.13	<0.001	231	38	26	-18
Brain stem	R	-	8.96	<0.001	235	2	-32	-2
Pallidum	L	-	7.96	<0.001	375	-16	0	8
Gyrus rectus	L	11	7.95	<0.001	81	-8	50	-20
Inferior frontal gyrus	L	47	7.62	<0.001	569	-34	22	-14
Superior frontal gyrus	L	6	7.36	0.001	101	-22	8	68
Pallidum	R	-	7.25	0.001	316	16	6	6
Inferior frontal gyrus	R	48	6.86	0.002	341	50	18	20
Precentral gyrus	R	6	6.84	0.002	97	50	6	48
Brain stem	L	-	6.77	0.002	112	-6	-28	-24
Precentral gyrus	L	6	6.62	0.003	227	-46	2	32
Inferior frontal gyrus	R	45	6.60	0.004	128	50	26	2
Supplementary motor area	L	6	6.42	0.005	50	-4	14	54
Superior parietal lobe	R	7	6.19	0.009	57	22	-64	52
Supplementary motor area	R	6	5.86	0.020	35	6	12	66
Amygdala*	L	-	4.85	0.001	180	-16	-2	-14
Amygdala*	R	-	4.87	0.001	199	34	2	-20
**Permit negative > view negative**
Amygdala*	L	-	3.77	0.014	3	-16	-2	-12

Results marked with an asterisk (*) are thresholded at *p* = 0.05, FWE-corrected for multiple comparisons for a region of interest; otherwise, all results are thresholded at *p* = 0.05, FWE-corrected for the whole brain, and limited to clusters with a spatial extent greater than *k* = 25 voxels. H, hemisphere; BA, Brodmann area, L, left; R, right. All *x, y, z* coordinates are in MNI space. Size is given in voxels (8 mm^3^).

### EXPLORATORY WHOLE-BRAIN ANALYSIS

**FIGURE 4:**
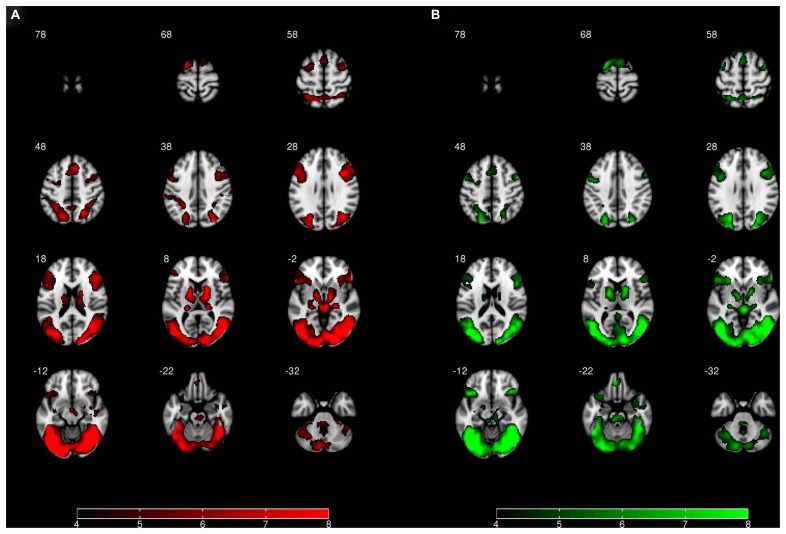
**Whole-brain activation for (A) the effect of “view negative” > “view neutral” and (B) the effect of “permit negative” > “view neutral.”** Image left is anatomical left. All statistical images represent *t*-values and are thresholded at 4 ≤ *t* ≤ 8.

In addition to our regions of interest analysis for the bilateral amygdala, we also conducted an exploratory whole-brain analysis. For this analysis, a comparison of the “view negative” vs. “view neutral” conditions at a threshold of *p* = 0.05 FWE revealed greater activation in the “view negative” condition with prominent clusters in the middle temporal gyrus, the right inferior frontal gyrus and the anterior cingulate cortex (see **Table 1**; **Figure 4**); peak differences were observed in the right middle temporal gyrus, being a part of a large cluster encompassing bilateral occipital and temporal areas. The reverse contrast revealed that no part of the brain showed greater activation in the “view neutral” than in the “view negative” condition.

A comparison of the “permit negative” vs. “view neutral” conditions revealed greater activation in the “permit negative” condition, which was most pronounced in the bilateral occipital, temporal, frontal, and cingulate cortex (see **Table 1**; **Figure 4**). Peak differences were observed in the fusiform gyrus, which was – similar to the previous comparison – part of an extended occipitemporal network. No part of the brain showed greater activation in the “view neutral” than in the “permit negative” condition.

Finally, a comparison of the “permit negative” vs. “view negative” conditions did not yield any significant results at the whole-brain level, corrected for multiple comparisons.

### CORRELATION ANALYSIS

**FIGURE 5:**
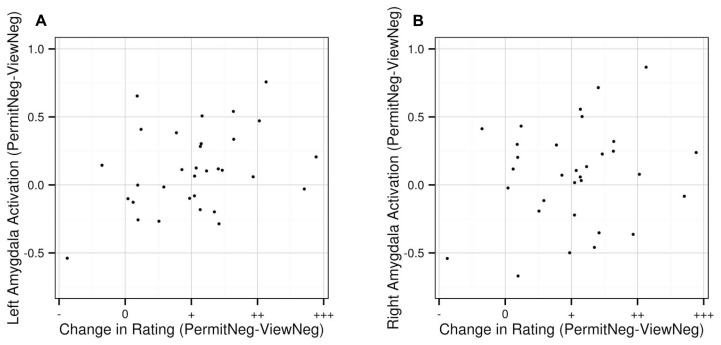
**Correlation between changes in subjective arousal ratings between “permit negative” and “view negative” and corresponding changes in activation the left (A) and right (B) amygdala, based on extracted values from the anatomically defined ROI volumes.** Values on the *x*-axis represent decreases (“permit negative” < “view negative”; indicated by “-”), no change (“permit negative” = “view negative”; indicated by “0”), and increases (“permit negative” > “view negative”; indicated by “+,” “++,” and “+++”).

As a result of an exploratory correlation analysis, we observed a positive relationship between changes in subjective arousal and changes in left amygdala activation between the “permit negative” and “view negative” conditions with a trend toward significance (ρ = 0.324, *p* = 0.071). There was no such effect for the right amygdala (ρ = 0.173, *p* = 0.342). The correlation indicates that for comparisons of “permit” and “view,” in general, a greater increase in subjective arousal is associated with a greater increase in activation in the left amygdala (**Figure 5**).

## DISCUSSION

The aim of the present study was to compare two commonly used baseline conditions for cognitive emotion regulation paradigms. We contrasted the instructions to passively view neutral and negative emotional stimuli, respectively, with an instruction to actively permit any feelings arising in response to emotionally negative stimuli. Subjective arousal ratings indicated that in the different experimental conditions stimuli were processed differently: as expected, negative stimuli yielded higher arousal ratings than neutral stimuli, and the “permit” instruction yielded higher ratings than the “view” instruction. Similarly, we observed differences in hemodynamic activation between negative and neutral stimuli as well as “view” and “permit” instructions. The pattern of these differences, however, was dependent on the specific contrasts: while activation differences between neutral and negative stimuli were present across large parts of the brain, including increased activation in the bilateral frontal, temporal, occipital cortex as well as subcortical areas for negative compared to neutral stimuli, an activation increase in the “permit negative” condition as compared to the “view negative” condition was only present for an analysis restricted to the amygdala.

The significant differences in subjective arousal ratings hint at the success of our emotion induction. At the neural level, extensive activation differences for comparisons between negative and neutral stimuli indicate that emotion effects are present beyond the expected amygdala activation and not necessarily limited to areas typically regarded as emotional brain areas, although such areas, e.g., the anterior cingulate cortex, were also present. In addition, however, many sensory and cognitive areas were also activated, such as the occipital or frontal cortex. This possibly reflects attentional feedback circuits (Sabatinelli et al., 2005; Vuilleumier, 2005), which may be triggered by emotion networks. Activation in cortical regions associated with higher cognitive function may also hint at the presence of automatic regulatory processes, which may be present even during seemingly simple tasks. This is consistent with the idea that emotional responses are not characterized by either the presence or the absence of emotional regulation, but rather a varying amount of emotional regulation, depending on situational or individual factors (Phillips et al., 2008). If that is the case, “passive viewing” might not be a fully adequate label for the mental state of the participants during this experimental condition.

The overall activation patterns for the “view negative” and “permit negative” conditions are similar: they are both characterized by extensive clusters of activation in the bilateral occipital, temporal, frontal, and cingulate cortex, and both engage the amygdala, when contrasted with the “view neutral” condition. From a neural perspective, the differences between these two conditions are rather quantitative than qualitative, and the commonalities are clearly larger than the differences. Differences between these two conditions, however, were evident in the respective subjective arousal ratings, and were also present in the left amygdala. It is possible that similar differences would also appear for ROI analyses in other regions of the brain; this, however, would require an independent experiment with a strong *a priori* hypothesis. There was also a lateralization of this effect, which we observed only in the left, but not right amygdala. It has been shown that the left amygdala is more frequently activated during emotional tasks (Baas et al., 2004) and more susceptible to emotional regulation instructions (Diekhof et al., 2011). Although not expected *a priori*, our results are in agreement with these reports.

Our *post hoc* finding of a correlation between changes in subjective arousal and changes in amygdala activation has to be regarded as an exploratory result and interpreted with caution. It may be taken as additional evidence for the validity of our experimental manipulation, indicating a difference between the “view negative” and “permit negative” instructions. Still, the distribution of the contrast estimates also shows that a robust amygdala increase has been lacking in some participants. While some variance in emotional responses across participants has to be expected, it might also be the case that some participants could not easily adhere to the experimental task, and that some emotional allowance was present during the passive viewing condition, or vice versa. Therefore, this observation suggests that instructions and/or training may benefit from further refinement.

A recent meta-analysis of amygdala activation has contrasted, among many other variables, passive viewing with explicit processing instructions such as explicit labeling, rating of the emotional stimulus, or feeling the emotion elicited by the stimulus (Costafreda et al., 2008). A key result was that increased cognitive demand leads to greater amygdala inhibition, which was also the case for a “feeling” instruction. At first sight, this seems to be a contradiction to our results. However, apart from the heterogeneity of samples, stimulus materials, and experimental contexts, the feeling instruction, in particular, included such diverse instructions as focusing on the self-relevance or trying to “react normally,” and therefore does not exactly correspond to our “permit” instruction. Furthermore, results of comparisons between and within studies are not directly comparable. This only holds if the respective control conditions as well as other experimental variables are comparable. In addition, given a link between emotional ratings and activation in emotional brain regions including the amygdala (Anders et al., 2004; Stark et al., 2007), it is unclear why participants should experience increased emotional arousal, as they reported in our study, but not engage – or even disengage – their amygdala at the same time. Finally, our post-scan ratings of task difficulty indicated that participants did not perceive the task to be particularly difficult.

This last consideration, together with the absence of strong frontal effects for the “permit negative” vs. “view negative” comparison, hints at an interpretation that cognitive effort may play a lesser role in our experimental paradigm. Therefore, it might be the case that increased effort and activation are not necessary for performing this particular task. This also suggests that the “permit” condition might be dissimilar to emotional up-regulation, which should involve more cognitive effort as well as increased activation in regulatory networks (Ochsner et al., 2004). It is possible that voluntarily permitting one’s emotions shares some characteristics with emotional up-regulation; however, up-regulation can be expected to have a much stronger effect, since there are some cognitive strategies that are part of up-regulation, but clearly no part of active permission (e.g., artificially exaggerating the emotional experience). Although it is a *post hoc* interpretation, it is also likely that our “permit” instruction does not necessarily involve affect labeling, which incorporates explicit instructions for high-level processing, and thus higher cognitive effort, and has been shown to lead to decreased amygdala activation (Lieberman et al., 2007; Payer et al., 2012).

In order to better understand the complex construct of emotion regulation, it has been suggested to reframe it in terms of its constituent sub-components (possibly including attentional and working memory processes, response inhibition, or conflict monitoring; Ochsner and Gross, 2005). To directly infer such cognitive components from complex emotion regulation paradigms, however, is not straightforward, since there is little experimental control on how participants actually implement a particular regulation strategy. Therefore, claims about cognitive sub-components are necessarily speculative, but it seems plausible to assume that attentional processes, possibly interoception, and possibly self-referential processes play a role for the active allowance of emotions.

As an example, the instructions to “attend to emotional stimuli,” “passively view” and to “attend to emotions elicited” differ in the degree to which attention is directed toward the emotional stimulus or the emotional response. This corresponds to differences in the amount of internal vs. external attention, which have been suggested to be two separable processes (Chun et al., 2011). We suggest that this also leads to differences in the amount of automatic emotion regulation, which, in turn, results in different levels of subjective arousal as well as amygdala activation. Directing attention toward the emotional stimulus is likely to stimulate deeper evaluation of emotion-evoking material. This can be assumed to lead to higher cognitive engagement, which has been shown to result in automatic down-regulation of the emotional response (Kalisch et al., 2006). In this case, less subjective arousal as well as less amygdala activation can be expected. Passive viewing grants higher degrees of freedom to the participants, and increased influence of habitual response tendencies might lead to increased variance in the emotional responses (Volokhov and Demaree, 2010). It can therefore be assumed to lead to less overall automatic down-regulation and higher subjective arousal as well as higher amygdala activation than the explicit attend condition. Active permission of the emotional response stimulates an internal focus of attention, which might reduce automatic down-regulation (Hutcherson et al., 2005). Therefore, for this condition relatively high (i.e., non-decreased) levels of arousal and amygdala activation in comparison to the other conditions can be expected. Taken together, both relative increases and decreases in amygdala activation as compared to the passive viewing instruction can be expected: stimulus-focused tasks are more difficult than passive viewing and could lead to a relative decrease, while response-focused tasks increase the focus on self-relevant aspects and might lead to a relative increase.

From a clinical perspective, these considerations and our results are in line with the idea that permitting or accepting an emotional state might be associated with greater distress in the short run but successful emotion regulation in the long run (Hayes et al., 1996). The so called “experiential avoidance” is seen as a dysfunctional transdiagnostic process which is successfully treated via exposure therapy or mindfulness therapy, during which the patients learn to tolerate and accept emotion experience in order to benefit from it. Generally, this includes a cognitive aspect (reduction of dysfunctional interpretation of emotion reaction) as well as an experiential aspect (actually accepting the physiological aspect of the emotion), which might both be addressed in a “permit” instruction.

A number of limitations of the current study need to be addressed. First, a recurrent issue for emotion regulation paradigms is whether or not to include trial-by-trial ratings: besides keeping participants actively engaged during the task, ratings ideally provide an immediate means of assessing the participants’ current state during each single trial of the experiment, thereby increasing the amount of experimental control. However, we cannot rule out the possibility that some participants may have responded according to their subjective hypotheses about the experiment, or may not have been able to accurately assess their emotional state consistently; this, however, is a general drawback of explicit emotion ratings (Stark et al., 2007). Further, in order not to increase the duration and complexity of each trial, we had to make a choice between arousal and valence ratings, and both would have been equally justifiable. Post-scan ratings, although not without disadvantages either, might have been useful for the simultaneous assessment of both measures. Physiological measures such as electrodermal activation, heart rate, or eye-tracking could certainly have supplemented our behavioral ratings, but we currently know of no other means that could have replaced them, despite their apparent shortcomings. Second, we did not include a mere “attend to stimulus” condition in our experimental design, but such a comparison would be needed in order to be able to test the above predictions about the influence of internal vs. external attention. Third, from a methodological point of view, a fully balanced factorial design, which contains one experimental factor for the stimulus (“neutral” vs. “negative”) and one factor for the task (“view” vs. “permit”), would be desirable for future studies, since this will allow a disambiguation of main and interaction effects. As a consequence of our incomplete design, the contrast “permit negative” vs. “view neutral” consists of the variation of two experimental factors, which limits its interpretability. While we assume that both factors contribute to the activation difference between the “permit negative” and “view neutral conditions,” we currently can only establish a stimulus effect for the “view” condition (“view neutral” vs. “view negative”) and an instruction effect for negative stimuli (“view negative” vs. “permit negative”). Since we initially doubted the psychological validity of “permit” instructions for neutral stimuli and weighted it against the issues of increased length and complexity, we refrained from implementing this condition. Still, it might be possible that even some seemingly neutral stimuli elicit emotional responses in some participants, and that emotional allowance might be applicable in such a case. In this regard, our retrospective view is not the same as our prospective view during the planning phase. Variations of our instructions or stimulus materials, however, could possibly allow for a factorial design, similar to experimental designs investigating the interaction of attention and emotion (Kanske and Kotz, 2011). Finally, the overall evidence of differences between the “view” and “permit” instructions, in this study, is not strong, both with regard to the strength and spatial extent of the effect. We did not expect the effect to be lateralized to the left hemisphere, and cannot determine yet how specific these effects are, i.e., to which extent other brain regions besides the amygdala would also show an effect of the experimental instruction. Therefore, an independent replication and extension of the results of this study would be desirable.

Several directions for future work can be derived from this study, both based on its results and its limitations. First, one goal for future work might be the further development of methods to validate the effects of the experimental manipulations, e.g., by using psychophysiological methods, by comparing trial-by-trial and post-scan ratings, and by assessing both valence and arousal, possibly using the “affect grid” (Russell et al., 1989) as a single behavioral measure. Together, this might alleviate the controversial issue of behavioral trial-by-trial ratings, which has not been satisfactorily solved in this study. Second, another goal might be the characterization of the affective and cognitive processes underlying the “permit” instruction. In particular, this involves investigating the contributions of attention, interoception, and similar subprocesses, which will also help to distinguish active emotional allowance from voluntary up-regulation of the emotional experience. Third, it would also be helpful to determine the effects of variations in task difficulty and cognitive effort, since both substantially influence emotional processing and regulation. Fourth, future studies should investigate if there are any contrast or carry-over effects between experimental conditions; in emotion regulation paradigms, verbal instructions are not always defined unambiguously, and processing during one experimental condition may depend on the processing during the other condition (e.g., participants may internally try to maximize the difference between two conditions). And finally, we do not know yet if specific forms of either baseline or regulation instructions work equally well for all individuals or under all circumstances, and it is also unclear whether or not our results generalize to positive emotions.

Despite these remaining open questions, the conceptual considerations and empirical evidence presented in this study suggest that there is no single optimal baseline condition for cognitive emotion regulation paradigms. The “view” and “permit” instructions are probably not exchangeable, and appear to have specific advantages and disadvantages: passive viewing may result in a lesser degree of experimental control and accordingly more variable emotional responses, but should be maximally dissimilar with regard to cognitive effort and associated neural activation when compared with any form of voluntary emotion regulation. Active permission has not yet been clearly distinguished from emotional up-regulation, and, also from a clinical perspective, might tap on similar mechanisms. It might, however, maximize the difference in activation between baseline and treatment conditions in emotion regulation paradigms. Accordingly, an optimal choice will depend on the experimental context: the “view” instruction might be a good choice for studies investigating differences between emotion regulation strategies, since passive viewing is not systematically related to effortful regulation; the emotional response itself, however, may be variable under this condition and possibly lower than its natural maximum. In contrast, the “permit” instruction might be better suited to demonstrate emotion regulation effects *per se*, because it appears to maximize the effect of the emotion induction, at the possible cost of introducing additional volitional processes. We therefore conclude that both strategies constitute suitable baseline conditions for studies of cognitive emotion regulation. Due to the importance of the experimental counterpart to the treatment condition, we suggest that this decision should be made consciously and carefully.

## AUTHOR CONTRIBUTIONS

Kersten Diers, Burkhard Brocke, Alexander Strobel, and Sabine Schönfeld designed the research; Kersten Diers and Fanny Weber performed the measurements; Kersten Diers and Alexander Strobel conducted the analysis; Kersten Diers, Fanny Weber, Burkhard Brocke, Alexander Strobel, and Sabine Schönfeld wrote the paper.

## Conflict of Interest Statement

The authors declare that the research was conducted in the absence of any commercial or financial relationships that could be construed as a potential conflict of interest.

## Footnotes

1 http://www.fil.ion.ucl.ac.uk/spm/software/spm8

2 http://www.nitrc.org/projects/wfu_pickatlas

3 http://marsbar.sourceforge.net

4 http://r-project.org
